# Two Metagenome-Assembled Genomes of Hydrogen-Dependent *Methanomassiliicoccales* Methanogens from the Zoige Wetland of the Tibetan Plateau

**DOI:** 10.1128/MRA.00021-21

**Published:** 2021-04-29

**Authors:** Juanli Yun, Wenbin Du

**Affiliations:** a State Key Laboratory of Microbial Resources, Institute of Microbiology, Chinese Academy of Sciences, Beijing, China; b Savaid Medical School, University of the Chinese Academy of Sciences, Beijing, China; Portland State University

## Abstract

Wetlands in the Tibetan Plateau play a crucial role in global carbon cycling. Here, we report the metagenome-assembled genomes (MAGs) of two hydrogen-dependent methanogens from the Zoige wetland of the Tibetan Plateau. The novel species belong to *Methanomassiliicoccales*, the seventh euryarchaeal methanogenic order.

## ANNOUNCEMENT

Methanogens are a group of archaea that control methane production in wetlands. Wetlands in the Tibetan Plateau are the major methane emission center in China (1). The genome research on uncultured methanogens in this extreme environment is of great importance in explaining the methane cycle in high-altitude wetlands.

Previously, extant methanogenic organisms were thought to belong exclusively to the phylum *Euryarchaeota* (2, 3), although more recently this assertion has been challenged by reports about the *Bathyarchaeota* (4, 5), *Verstraetearchaeota* (3, 6), and newly discovered *Cyanobacteria* (7, 8) phyla. Methanogens from the *Methanomassiliicoccales* order are called the seventh order of methanogens and are widely distributed in various environments (9). Here, we announce two metagenome-assembled genomes (MAGs) of novel *Methanomassiliicoccales* species with medium completeness.

Two sediment cores from the Flower Lake National Reserve of the Zoige wetland (102°52′E, 33°56′N) were sampled using sampling equipment (10 cm in diameter). The sampling site was water saturated, and the standing water depth was about 20 cm. Sediment cores were mixed thoroughly and kept at −80°C before use. DNA was extracted from the two sediment samples using the FastDNA spin kit for soil (MP Biomedicals, Cleveland, OH, USA) following the manufacturer’s instructions. A shotgun library was prepared with the NEBNext kit. Sequencing was completed on an Illumina HiSeq 2 × 150-bp platform. The average amount of metagenomic raw data for each sample was approximately 30 Gbp.

Sequencing quality for each sample was checked with FastQC (v.0.11.8) (10), and low-quality reads were trimmed using Trimmomatic (v.2.1.7) (11). Clean data were assembled individually using MEGAHIT (v.1.0) (12). To obtain MAGs, sequencing reads for each sample were mapped to the contigs using Bowtie 2 (v.2.2.5) to obtain differential coverage of each sample (13); genome binning was conducted based on these differential coverage files with MetaBAT (14) using a 1,000-bp contig cutoff value. The completeness and contamination of the MAGs were estimated using CheckM (v.1.1.2) (15). MAGs containing *mcrA* genes were selected by GraftM (v.0.13.1) (16) and annotated using Prokka (v.1.14.6) (17). rRNA coding regions (16S and 23S) of MAGs were predicted with Barrnap (https://github.com/tseemann/barrnap). Default parameters were used for all software unless otherwise specified.

The two MAGs obtained in this study have genome sizes of 1.05 Mb (bin 47) and 1.67 Mb (bin 107), and the genome completeness values were 67.7% with 2.42% contamination and 86.6% with 4.84% contamination, respectively (Table 1). The MAGs were both identified as *Methanomassiliicoccales* strains according to the *mcrA* gene taxonomy assignment, with 81.3% and 83.8% similarities to the *Massiliicoccales* Lake Pavin MAG according to an online BLAST search of the NCBI nucleotide database of *mcrA* genes (18).

**TABLE 1:** Sequence statistics and metagenomic binning statistics for each archaeal genome

Parameter	Data for:	
	Bin 47	Bin 107
Collection site	Zoige wetland (China)	Zoige wetland (China)
No. of reads for assembly	75,418,769	93,013,493
Total length (bp)	1,057,695	1,674,866
No. of contigs	221	189
GC content (%)	58.2	57.3
No. of coding regions	1,139	1,701
*N*_50_ (bp)	5,726	10,906
No. of 16S rRNA genes	None	None
Completeness^*a*^ (%)	67.7	86.6
Contamination^*a*^ (%)	2.42	4.84

a The completeness and contamination values are based on the CheckM estimations.

The two MAGs contain all genes required for hydrogen-dependent reduction of methanol to methane, as proposed for other *Methanomassiliicoccales* strains (18). This announcement provides the basis for isolating this clade from environments.

### Data availability.

The *Methanomassiliicoccales* genome sequences have been deposited in GenBank under the accession numbers JACXTO000000000 and JACXTP000000000. The versions described here are the first versions. All metagenomic data generated from this announcement are available under BioProject number PRJNA644254. Metagenomic bins can be found under BioSample numbers SAMN15455434 and SAMN15455435.
